# A Capacitive Humidity Sensor Based on an Electrospun PVDF/Graphene Membrane

**DOI:** 10.3390/s17051009

**Published:** 2017-05-03

**Authors:** Daniel Hernández-Rivera, Grissel Rodríguez-Roldán, Rodrigo Mora-Martínez, Ernesto Suaste-Gómez

**Affiliations:** Electrical Engineering Department, Bioelectronics Section, Centro de Investigación y de Estudios Avanzados del IPN, Av. IPN 2508, Col. San Pedro Zacatenco, Mexico City 07360, Mexico; hernandezr@cinvestav.mx (D.H.-R.); grodriguezr@cinvestav.mx (G.R.-R.); rmora@cinvestav.mx (R.M.-M.)

**Keywords:** humidity, sensor, PVDF, graphene, electrospinning

## Abstract

Humidity sensors have been widely used in areas such as agriculture, environmental conservation, medicine, instrumentation and climatology. Hydrophobicity is one of the important factors in capacitive humidity sensors: recent research has shown that the inclusion of graphene (G) in polyvinylidene fluoride (PVDF) improves its hydrophobicity. In this context, a methodology to fabricate electrospun membranes of PVDF blended with G was developed in order to improve the PVDF properties allowing the use of PVDF/G membrane as a capacitive humidity sensor. Micrographs of membranes were obtained by scanning electron microscopy to analyze the morphology of the fabricated samples. Subsequently, the capacitive response of the membrane, which showed an almost linear and directly proportional response to humidity, was tested. Results showed that the response time of PVDF/G membrane was faster than that of a commercial DHT11 sensor. In summary, PVDF/G membranes exhibit interesting properties as humidity sensors.

## 1. Introduction

Currently, humidity sensors are widely used in diverse areas such as agriculture, environmental conservation and medicine. Specifically, in the medical field, these sensors are used in respiratory equipment, incubators, sterilizers and biological products, where humidity control is very important [1,2,3,4]. Besides, there is an increasing demand for devices with optimum properties for different applications, in aspects such as cost, compatibility, sensibility and response time.

One of the most common ways to express this parameter is through relative humidity (RH). RH is defined as the ratio, expressed as a percentage, of the amount of humidity present in a gas, at a certain temperature, to the maximum humidity level that the gas can hold at that same temperature [1,4].

The humidity sensors research has generated a wide variety of devices composed of polymers, ceramics, blended materials, graphene (G)-based materials, and others [2,5,6,7,8,9,10,11,12,13,14]. The humidity level is obtained by measurement of either the electrical resistance or capacitance of the sensing material [4]. Capacitive humidity sensors exhibit several advantages in comparison with resistive humidity sensors, such as linear responses, fast response times and low hysteresis [4]. The characteristics of capacitive humidity sensors are widely variable due to the different manufacturing processes used; some examples of capacitive humidity sensors present a sensitivity in the range from 0.005 pF/% RH to 0.077 pF/% RH, with response times between 4 s and 47 s [15,16,17].

These kinds of humidity sensors are mostly made of hydrophobic materials, avoiding the storage of water molecules in the sensing material and letting water vapor flow freely. The formation of clusters of absorbed water molecules leads to hysteresis; this is a serious disadvantage in capacitive humidity sensors [1,4,18].

Polymeric materials have been widely used as sensing materials due to their advantages of low cost, easy preparation, excellent stability and good mechanical properties [7]. Nanofibers fabricated by electrospinning provide a rough surface this way, increasing hydrophobicity, an ideal property in capacitive humidity sensors [19,20]. In the electrospinning process, a high voltage induces a charged jet of a polymer solution; this elongated jet is moved toward a collector plate where fibers are randomly deposited. The positive electrode is placed in the solution and the collector plate is grounded [21,22]. Parameters such as feed rate, voltage, distance between tip and collector and others can modify the morphology of fibers [22,23,24].

The incorporation of nanoparticles into the polymeric solution is another method for changing membrane properties [25]. Graphene (G) is a two dimensional monoatomic thick sheet composed of sp^2^ carbon atoms arranged in a hexagonal lattice and it is the elementary structure of graphite. In recent years, there has been an increasing interest in G because of its excellent electrical conductivity, strong mechanical strength, thermal conductivity and ease of functionalization. Therefore, G-based materials are highly attractive [26,27]. G has been used as an additive in polymers and other materials in order to modify their electrical, mechanical, optical and thermal properties [28]. Recent research has shown that the inclusion of G in polymeric materials improves their hydrophobicity [19,25,29].

PVDF has received especial attention among a wide variety of polymers due to its thermal and chemical stability, mechanical and ferroelectric properties which can be easily conformed to complex surfaces [3,8,30,31,32,33,34,35]. Moreover, its biocompatibility makes PVDF sensors desirable candidates to be used in biomedical applications [31,33,36]. Besides its sensing capability, PVDF is also used in structural health monitoring systems such as pressure sensors and many other applications such as temperature and humidity sensing.

It has been shown that the RH of the surrounding atmosphere can modify the dielectric properties of electrospun PVDF [8]. The aim of this paper is to analyze the dielectric response of electrospun PVDF/G membrane as a capacitive humidity sensor. Scientific research has proven that the incorporation of G into the PVDF enhances its hydrophobicity; this could result in a humidity sensor with fast response time and low hysteresis.

## 2. Materials and Methods

### 2.1. Preparation of PVDF/G Solution

This paper focus on a mixture of PVDF with G in order to obtain a humidity sensor with different characteristics with regard to sensitivity, response time and hysteresis. Recently, studies have shown that mechanical and thermal properties of PVDF are improved by the addition of G [19,29,37,38].

PVDF is dissolved in polar solvents such as *N*,*N*-dimethylformamide (DMF) at room temperature. This kind of polymer solution was used to fabricate nanofibers by electrospinning. Rough and porous structures are attractive elements for humidity sensors because they allow water vapor to pass through and are hydrophobic [19,20].

PVDF powder and DMF were purchased from Sigma Aldrich (Saint Louis, MO, USA). Ultra-high Concentration Dispersion of Graphene Nanoplatelets was purchased from Graphene Supermarket (Calverton, NY, USA). Two solutions were prepared: PVDF/DMF and PVDF/G/DMF. The PVDF/DMF solution with a 20 wt % composition was prepared by dissolving PVDF powder in DMF at 60 °C, with the purpose of ensuring a homogeneous mixture. PVDF/G/DMF solution with 20 wt % composition was prepared as follows: PVDF powder and G were thoroughly weighted in order to obtain PVDF/G membranes with 0.5 wt % composition, which was chosen based on the investigation about hydrophobic PVDF/G membranes [19,25,29]. The method to prepare these solutions is based on actual research [37]. Firstly, G was dispersed in DMF by using an ultrasound dispersion machine during three 30-min sessions to generate a homogenous solution. Separately, PVDF powder was dissolved in DMF at 60 °C during two hours with the aim of ensuring a homogeneous mixture. After, PVDF/DMF and G/DMF solutions were mixed, stirred and sonicated during two 30-min sessions. Finally, a uniform solution with an appropriate concentration (20 wt %) for electrospinning was obtained.

### 2.2. Membrane Fabrication

**Figure 1:**
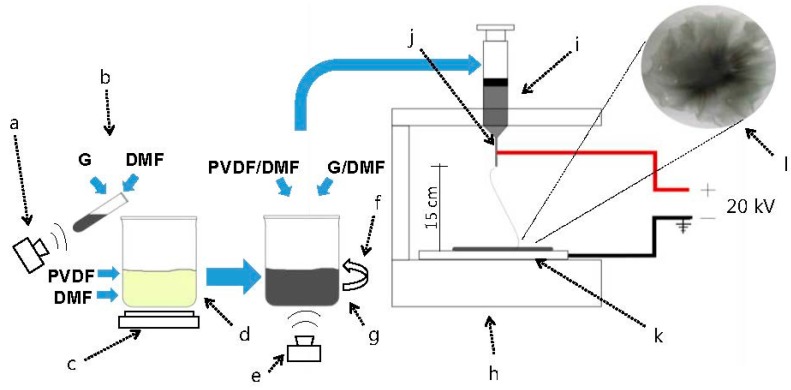
Fabrication process: (**a**) Sonication; (**b**) G/DMF solution; (**c**) Electric heater; (**d**) PVDF/DMF solution; (**e**) Sonication; (**f**) Stirring; (**g**) PVDF/G/DMF solution; (**h**) electrospinning equipment; (**i**) needle; (**j**) sysringe tip; (**k**) collector plate and (**l**) electrospun membrane.

**Figure 2:**
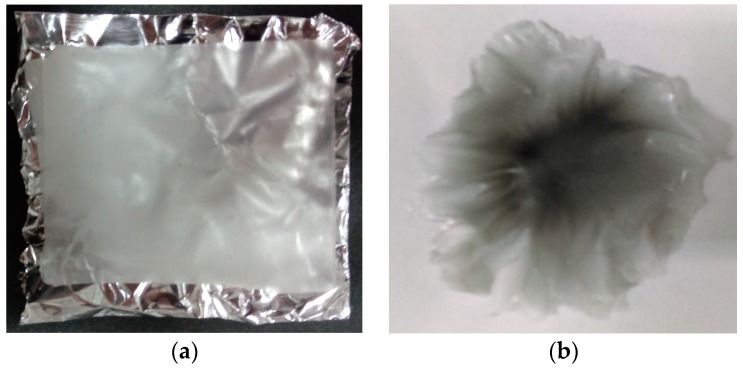
(**a**) PVDF and (**b**) PVDF/G membranes.

PVDF/G/DMF and PVDF/DMF solutions were used to fabricate membranes by electrospinning as illustrated in Figure 1. The electrospinning process was performed with the using of a 20 kV DC voltage, a syringe (volume of 3$\;{\mathrm{cm}}^{3}$, hypodermic needle) and an aluminum collector plate (15 cm × 10 cm), during 40 min at a feeding rate of 2.5 mL. The distance between the syringe tip and the collector plate was 15 cm, which was chosen to obtain nanometric fibers and membranes with a useful area for the sensing application. Finally, PVDF and PVDF/G membranes were obtained as shown in Figure 2.

### 2.3. Experimental Tests

**Figure 3:**
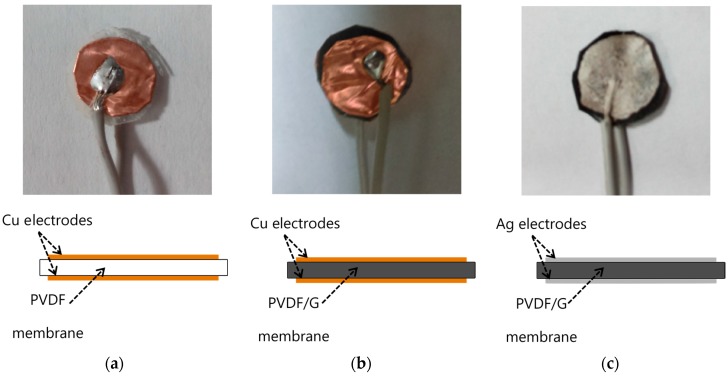
(**a**) PVDF-Cu; (**b**) PVDF/G-Cu and (**c**) PVDF/G-Ag samples.

In order to perform a morphological analysis, micrographs of membranes were acquired by a JSM-6360LV scanning electron microscope (JEOL, Akishima-shi, Japan). Circular samples of 1 cm diameter were obtained from the PVDF and PVDF/G electrospun membranes for the purpose of testing the behavior of these materials. The average thickness of PVDF and PVDF/G membranes was around 124.4 µm and 127 µm, respectively. Finally, copper tape electrodes were placed on both faces of the PVDF (PVDF-Cu) and PVDF/G (PVDF/G-Cu) samples. The choice of the electrodes for the samples depends on the membrane morphology because the porosity can allow that the conductive material is deposited inside the membrane. Taking into consideration the morphology of PVDF/G membrane, other sample was tested (PVDF/G-Ag), in this case a layer of silver paint was used as the electrode. The samples with electrodes are illustrated in Figure 3.

This paper will focus on a PVDF/G membrane as a humidity sensor; hence the humidity dependence of the dielectric constant was tested. If samples are used as the dielectric material in a parallel plate capacitor, the dielectric constant of electrospun samples shows a directly proportional behavior with respect to capacitance of the condenser [8,33]. Therefore, capacitive impedance measurements of PVDF-Cu, PVDF/G-Cu and PVDF/G-Ag samples were used to know the humidity dependence of the dielectric constant.

### 2.4. Humidity Sensor Tests

**Figure 4:**
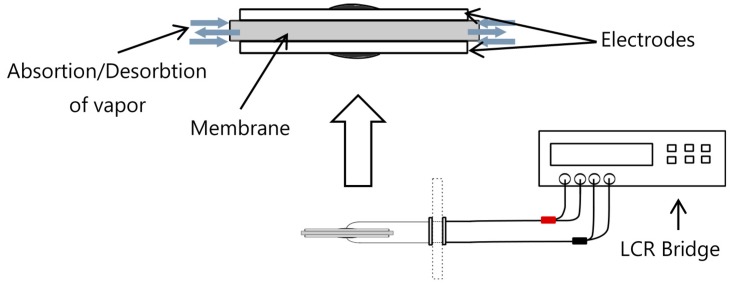
Humidity sensing mechanism.

Considering that samples were fabricated with electrospinning, the sensing principle of the proposed sensor is based on the dielectric constant change of membranes (measured with a LCR bridge) due to water vapor inside fibrous structure of these samples as shown in Figure 4.

In order to measure the response of the PVDF-Cu, PVDF/G-Cu and PVDF/G-Ag membranes to relative humidity changes, the samples were placed inside an insulation chamber of volume 0.018472 m^3^ and capacitive impedance was measured with a LCR bridge (HM818- Rohde & Schwarz, Munich, Germany) at 1 kHz for a varying humidity between 35 and 90% in concentration RH at room temperature (25 °C ± 1 °C).

**Figure 5:**
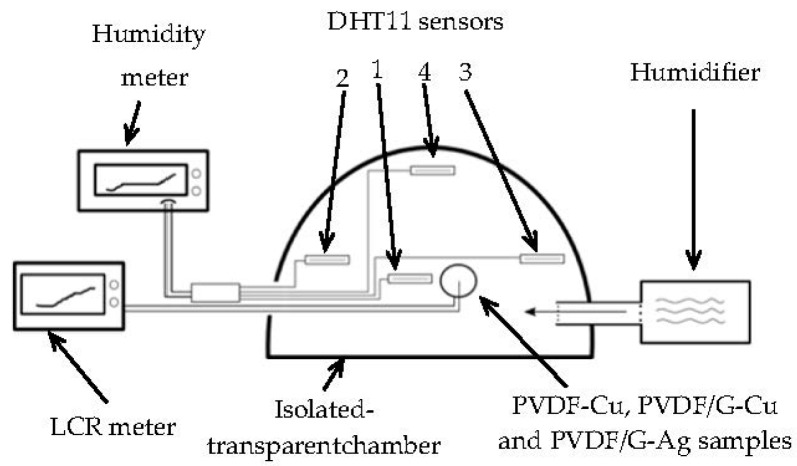
Experimental setup for the humidity tests.

Four commercial DHT11 humidity sensors (Aosong, Guangzhou, China) with the specifications listed in Table 1 were also introduced in the insulated chamber to establish the homogeneity of the humidity inside the chamber. The diagram of Figure 5 shows the way the tests were done.

## 3. Results

### 3.1. Morphological Analysis

**Figure 6:**
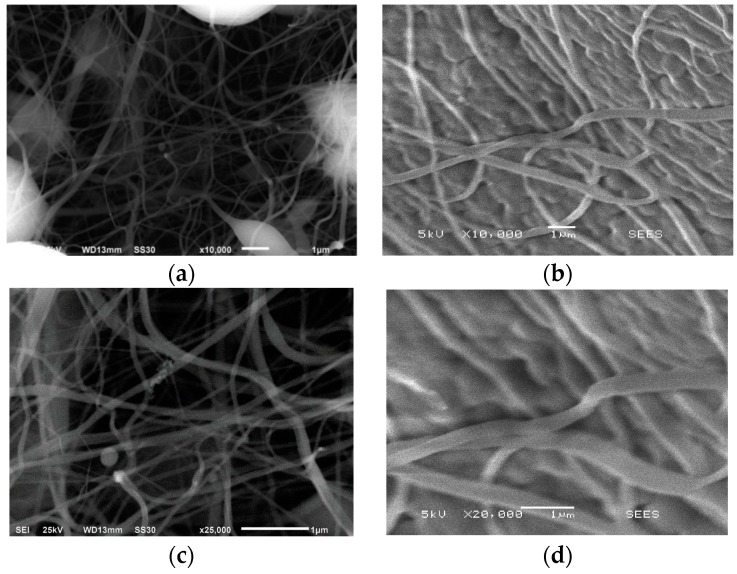
Micrographs of (**a**,**c**) PVDF at magnification 10,000×, 25,000× and (**b**,**d**) PVDF/G membranes at magnification 10,000×, 20,000×.

Micrographs of membranes, acquired by the JEOL JSM-6360LV scanning electron microscope are shown in Figure 6. PVDF fibers with diameters ranging from 50 nm to 700 nm and PVDF/G fibers with diameters ranging from 130 nm to 600 nm were achieved. On the other hand, PVDF/G membrane tends to have a lower total porosity than PVDF membrane.

### 3.2. Humidity Response

**Figure 7:**
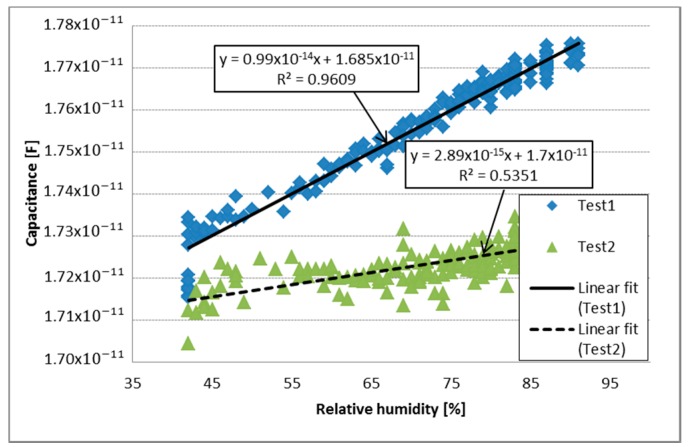
Capacitive response to relative humidity changes of PVDF.

Two humidity characterization tests (Test 1 and Test 2 for each sample) were done to analyze the humidity responses of PVDF-Cu, PVDF/G-Cu and PVDF/G-Ag samples; a comparative test between each sample and the commercial DHT11 humidity sensor was also performed to examine the response time to humidity drop. Taking into account that the humidity changes for different points inside chamber, the humidity measurements were acquired by the DHT11 nearest to the membrane. Firstly, PVDF membrane with copper electrodes was tested twice (Test 1 and Test 2) to analyze the behavior of PVDF membrane without G to relative humidity changes from 40% to 90%. The capacitive response to relative humidity changes can be seen in Figure 7. A linear fitting was also calculated for Test 1 and Test 2, presenting a coefficient of determination of 0.9609 and 0.5351 respectively, and a normalized root-mean-square error (NRMSE) of 5.25% for Test 1 and 16.34% for Test 2. Analyzing the graphs it can be observed that the response of each test (Test 1 and Test 2) is notably different, moreover, noise is present.

**Figure 8:**
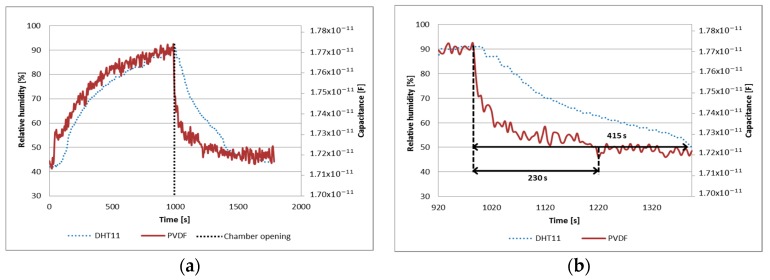
(**a**) Comparative response time and (**b**) response time to humidity drop of PVDF and DHT11.

Subsequently, comparative tests between PVDF membrane and DHT11 sensor were done. In this case, the response of the sensors was measured when humidity level drops rapidly inside transparent chamber (i.e., when the chamber is opened). The graph of Figure 8a shows the response time of both sensors and Figure 8b exhibits the response time of the sensors when RH concentration drops from 90% to 50%.

**Figure 9:**
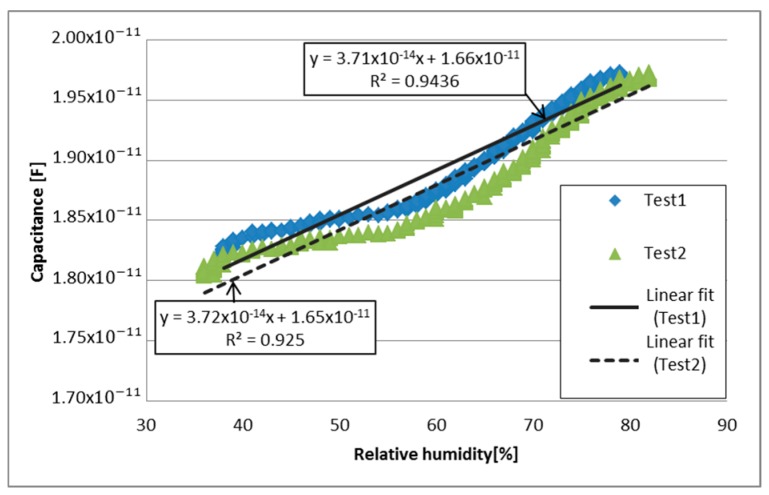
Capacitive response to relative humidity changes of PVDF/G-Cu.

The next test was performed with PVDF/G-Cu membrane. The capacitive response to relative humidity changes from 35% to 85% can be seen in Figure 9. A linear fitting was calculated for Test 1 and Test 2, presenting a coefficient of determination of 0.9436 and 0.925 respectively, and a NRMSE of 7.56% for Test 1 and 9.36% for Test 2. Unlike PVDF membrane, in this case it can be observed that the response of each test is similar and low noise is present.

A comparative test between PVDF/G-Cu membrane and DHT11 sensor when humidity level drops rapidly inside the chamber (i.e., when the chamber is opened) was also performed.

**Figure 10:**
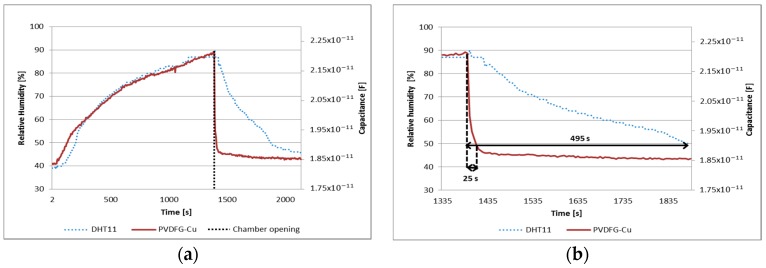
(**a**) Comparative response time and (**b**) response time to humidity drop of PVDF/G-Cu and DHT11.

The graph of Figure 10a shows the response of both sensors along the time, while Figure 10b exhibits the response time of both sensors when the RH drops from 90% to 50%. It is notorious that the response time of PVDF/G-Cu membrane is faster than the shown by the commercial sensor DHT11.

**Figure 11:**
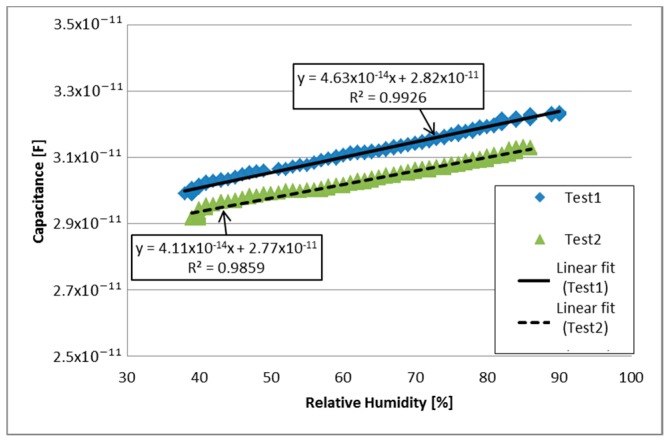
Capacitive response to relative humidity changes of PVDF/G-Ag.

One last test was performed using PVDF/G-Ag sample. Capacitive response to relative humidity changes can be seen in Figure 11. A linear fitting was also calculated for test 1 and test 2, presenting a coefficient of determination of 0.9926 and 0.9859 respectively, and a NRMSE of 2.51% for Test 1 and 3.16% for Test 2. In this case the response was similar to PVDF/G-Cu sample; it can be observed that the response of each test is similar and low noise is present, however, the capacitive impedance of this sample is greater than the impedance of PVDF/G-Cu sample.

**Figure 12:**
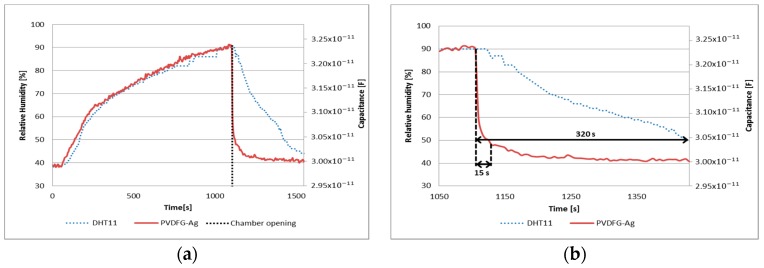
(**a**) Comparative response time and (**b**) response time to humidity drop of PVDF/G-Ag and DHT11.

Finally, comparative response times, between PVDF/G-Ag membrane and DHT11 sensor, were determined when humidity level drops rapidly inside chamber (i.e., when the chamber is opened). The graph of Figure 12a shows the response of both sensors along time and Figure 12b exhibits the response time of the sensors when the humidity concentration dropped from 90% to 50%. Similarly, as it was observed for the PVDF/G-Cu membrane, the response time of PVDF/G-Ag membrane is also faster than the shown by the commercial sensor DHT11.

Table 2 lists some previously obtained characteristics of PVDF-Cu, PVDF/G-Cu and PVDF/G-Ag as a way of contrast the results of the humidity tests.

## 4. Discussion

Analyzing the results, it can be observed that dielectric properties of the three fabricated samples are influenced by the RH. The capacitive response of the membranes showed an almost linear and directly proportional behavior with respect to humidity. The response time of the PVDF-Cu sample was 1.8 times that of the DHT11 sensor and had undesirable behavior regarding repeatability and noise, on the other hand, the Test 1 exhibited an almost linear behavior (R^2^ = 0.9609) with a NRMSE of 5.25%, however, the Test 2 showed a poor linear fit ($R^{2}=0.5351$) with a NRMSE of 16.34%. It could be caused by a widely variety of elements such as the design of electrodes used, that water molecules were stored in the sensing material, the morphology of electrospun membrane, among others; accordingly new tests must be proposed with the purpose of obtaining more data about the behavior of the PVDF sample.

On the other hand, PVDF/G membranes exhibited other interesting properties such as similar responses to each test (repeatability) and low noise in the acquired signals, however, it was noticeable that there was a difference in the magnitude of the capacitance between the samples. The sensitivity of PVDF/G-Cu and PVDF/G-Ag were better than PVDF sample as can be seen in Table 2 and similar to other reported designs [15,16,17], however, the capacitance of PVDF/G-Ag was greater than the PVDF/G-Cu sample under the same conditions, possibly due to the type of electrodes used, since silver paint can create a better contact with the membrane surface than copper tape. As seen in Figure 10 and Figure 12, the response time of PVDF/G samples was rather than the observed for the DHT11 sensor when the relative humidity dropped from 90% to 50%; PVDF/G-Cu membrane and PVDF/G-Ag membrane were 19.8 times and 21.3 times that of the DHT11 sensor, respectively.

PVDF/G samples have a faster recovery time than the commercial sensors; this phenomenon can indicate that there no significant formation of clusters results from the adsorption of water molecules by the sensing material, which could cause hysteresis and low response time. A reliable hysteresis measurement should be done in future research to confirm that there was no water molecules absorbed by PVDF/G samples.

Despite the similar behavior of the PVDF/G membranes, analyzing Figure 9 and Figure 11, PVDF/G-Ag sample exhibited a more linear behavior ($R^{2}=0.9926,{\;R}^{2}=0.9859$) with respect to humidity than the PVDF/G-Cu ($R^{2}=0.9436,{\;R}^{2}=0.925$), moreover, the normalized root mean square error for PVDF/G-Ag sample was 2.51% for Test 1 and 3.16% for Test 2, unlike of the linear fit of PVDF/G-Cu (7.56% for Test 1 and 9.35% for Test 2). These differences can only be attributed to the type of electrodes used in the tests, inasmuch as the two samples were obtained from the same membrane.

## 5. Conclusions

In this paper, it was possible to apply a methodology to fabricate PVDF/G membranes by electrospinning and use PVDF-Cu, PVDF/G-Cu and PVDF/G-Ag samples as capacitive humidity sensors. The sensing principle of the proposed sensor was based on the dielectric constant change of membranes due to water vapor inside fibrous structure of these samples. The morphology of membranes and the addition of G were used to modify the hydrophobicity of PVDF, which is an important factor for capacitive humidity sensors. The PVDF membrane can function as capacitive humidity sensor, however, the design of the PVDF capacitive humidity sensor must be improved in order to obtain good response time, better sensitivity, repeatability and low noise. PVDF is commonly used in different biomedical applications due to its biocompatibility, piezoelectric properties and the facility to be cast or molded by different techniques.

On the other hand, based on results, the sensitivity and linearity of PVDF/G samples were interesting and the response time was better than that of the commercial DHT11 sensor and the PVDF sample. A good response of PVDF/G as a humidity sensor was thus obtained by using the electrospinning technique and adding graphene, nevertheless, although scientific research has proven that the incorporation of G into the PVDF enhances its hydrophobicity, other tests such as hydrophobicity tests, hysteresis measurements and repeatability should be done in future work to know the mechanisms involved in the response of PVDF/G samples and then design sensors with improved features. PVDF/G membranes not only work as humidity sensors, but can also be used for temperature, light and pressure sensing due to their ferroelectric properties. Other possible applications could be filtration, growth of living tissues and the manufacture of prosthesis.

## Figures and Tables

**Table 1:** Sepecifications of the DHT11 humidity sensor.

Accuracy	Resolution	Response Time	Hysteresis	RH Range
±4% RH	1% RH	10 s	±1% RH	20% to 90%

**Table 2:** Characteristics of PVDF-Cu, PVDF/G-Cu and PVDF/G-Ag.

Sample	Sensitivity	Coefficient of Determination (*R*^2^)	Normalized Root Mean Square Error (NRMSE)	Response Time When RH Drops from 90% to 50% *
PVDF-Cu	Test 1: 0.0099 pF/% RH	Test 1: 0.9609	Test 1: 5.25%	1.8
	Test 2: 0.0028 pF/% RH	Test 2: 0.5351	Test 2: 16.34%	
PVDF/G-Cu	Test 1: 0.0371 pF/% RH	Test 1: 0.9436	Test 1: 7.56%	19.8
	Test 2: 0.0372 pF/% RH	Test 2: 0.925	Test 2: 9.35%	
PVDF/G-Ag	Test 1: 0.0463 pF/% RH	Test 1: 0.9926	Test 1: 2.51%	21.3
	Test 2: 0.0411 pF/% RH	Test 2: 0.9859	Test 2: 3.16%	

* Times the DHT11 response.
